# Implementing a QCancer risk tool into general practice consultations: an exploratory study using simulated consultations with Australian general practitioners

**DOI:** 10.1038/bjc.2015.46

**Published:** 2015-03-03

**Authors:** P P-C Chiang, D Glance, J Walker, F M Walter, J D Emery

**Affiliations:** 1 General Practice and Primary Health Care Academic Centre, University of Melbourne, 200 Berkeley Street, Carlton, Victoria 3053, Australia; 2 Centre for Software Practice, University of Western Australia, Crawley, Western Australia 6009, Australia; 3 General Practice, School of Primary Aboriginal and Rural Health Care, University of Western Australia, Crawley, Western Australia 6009, Australia; 4 The Primary Care Unit, Institute of Public Health, University of Cambridge School of Clinical Medicine, Box 113, Cambridge Biomedical Campus, Cambridge CB2 0SR, UK

**Keywords:** cancer, cancer risk assessment, primary care, early diagnosis, general practitioners, decision support

## Abstract

**Background:**

Reducing diagnostic delays in primary care by improving the assessment of symptoms associated with cancer could have significant impacts on cancer outcomes. Symptom risk assessment tools could improve the diagnostic assessment of patients with symptoms suggestive of cancer in primary care. We aimed to explore the use of a cancer risk tool, which implements the QCancer model, in consultations and its potential impact on clinical decision making.

**Methods:**

We implemented an exploratory ‘action design' method with 15 general practitioners (GPs) from Victoria, Australia. General practitioners applied the risk tool in simulated consultations, conducted semi-structured interviews based on the normalisation process theory and explored issues relating to implementation of the tool.

**Results:**

The risk tool was perceived as being potentially useful for patients with complex histories. More experienced GPs were distrustful of the risk output, especially when it conflicted with their clinical judgement. Variable interpretation of symptoms meant that there was significant variation in risk assessment. When a risk output was high, GPs were confronted with numerical risk outputs creating challenges in consultation.

**Conclusions:**

Significant barriers to implementing electronic cancer risk assessment tools in consultation could limit their uptake. These relate not only to the design and integration of the tool but also to variation in interpretation of clinical histories, and therefore variable risk outputs and strong beliefs in personal clinical intuition.

Primary care clinicians have a key role in diagnosing people with symptomatic cancer (Emery *et al*, 2014), but this can be challenging because the symptoms of many cancers are common in the community and overlap with more prevalent benign conditions (Lyratzopoulos *et al*, 2012; Stapley *et al*, 2012). Some tumour types are more difficult to diagnose than others in primary care: patients with lung, pancreatic and stomach cancer and myeloma are significantly more likely to pay multiple visits to their general practitioner (GP) before referral compared with patients with breast or endometrial cancer (Lyratzopoulos *et al*, 2012). Further adding to the complexity is that common symptoms are frequently associated with more than one type of cancer (Hippisley-Cox and Coupland, 2013b). Delays in primary care can contribute to later cancer diagnosis (Neal, 2009) with potential effects on prognosis, intensity of treatment and negative impacts on the quality of life (Singh *et al*, 2007). Delayed cancer diagnosis is one of the most common, harmful and costly types of diagnostic error in ambulatory care settings (Gandhi *et al*, 2006; Singh *et al*, 2009, 2010).

A recent systematic review of patient safety strategies targeted at reducing diagnostic errors by primary care clinicians found the strongest evidence for technology-based interventions such as computer-assisted diagnostic aids, decision support algorithms, text messages and pager alerts and adaptations to testing equipment (McDonald *et al*, 2013). In this study, we explore the feasibility of implementing a risk assessment tool that applies the ‘QCancer' cancer risk prediction model in general practice (Hippisley-Cox and Coupland, 2013a, 2013c). The model provides specific risks of different cancers according to combinations of baseline risk factors (BMI, smoking, family history and alcohol), current symptoms and specific clinical conditions. We chose to implement the QCancer model because it predicts the risk for 10 different types of cancers, many of which often present with vague and common and overlapping symptoms such as colorectal, gastro-oesophageal, lung, haematological, renal, pancreatic and ovarian cancer. The QCancer model has been externally validated and is one of the models applied in electronic cancer decision support (eCDS) (Dikomitis *et al*, 2014) evaluated in over 500 general practices in the United Kingdom; it is currently being more extensively implemented. In Australia there is interest in the use of cancer risk assessment tools, potentially as part of establishing fast-track referral routes in public hospitals (Emery *et al*, 2013). However, little is known about how cancer risk assessment tools might be used in primary care and what the challenges are to widespread implementation and uptake.

## Materials and methods

We used an ‘action design' method, in which practitioners used the prototype QCancer software (D Glance, University of Western Australia, Crawley, Australia) at their workplace to inform necessary changes to the programme and explore its potential utility (Timpka *et al*, 1994). The action design method meant that we modified specific aspects of the software interface during the study based on findings from initial simulated consultations. Although major modifications were beyond the scope of this project, we altered the way diagnostic guidance was highlighted so that GPs were more likely to access it, and reduced the prominence of the Disclaimer section. The overall research study was set within the Medical Research Council framework for the development and evaluation of complex interventions to improve health (Medical Research Council, 2008). This initial exploratory research was designed to develop the intervention and explore the context in which it would be implemented to inform decisions about subsequent studies in actual clinical settings (Campbell *et al*, 2007).

### Study participants

A purposive sample of GPs was recruited to cover a range of age, gender, years of clinical experience and geographical location within Victoria, Australia. General practitioners were identified from the Victorian Primary Care Practice-Based Research Network, from practices involved with the education and training at the University of Melbourne and from other research-naive practices. Recruitment continued until thematic saturation was reached when interviews yielded no further new themes, as determined by the two researchers who conducted all the research interviews. Participating GPs received $300 to compensate for their time.

### The QCancer risk tool

**Figure 1:**
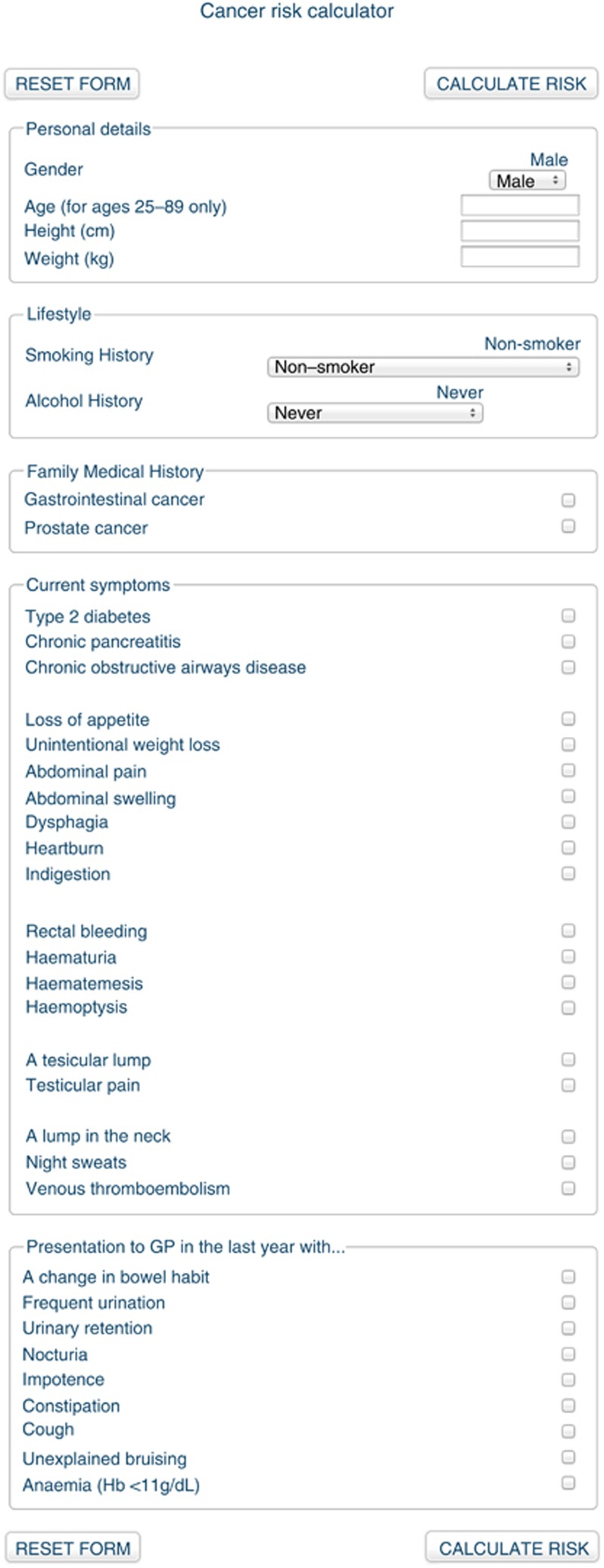

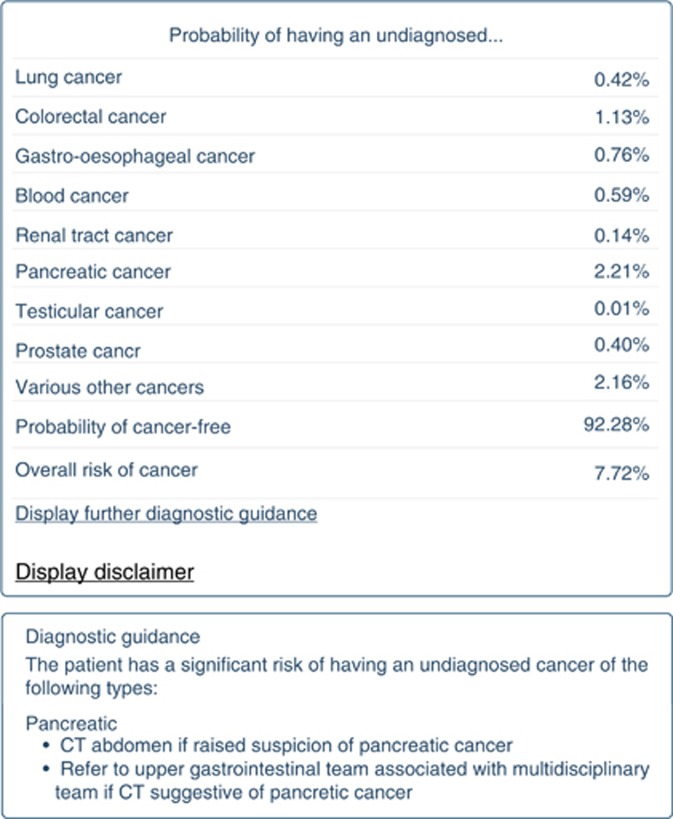
Screenshots of QCancer risk tool.

For this study the QCancer model was implemented into a specific web-based version, with the interface designed as a simple, single browser page (Figure 1). The source code for the QCancer risk model was provided by ClinRisk Ltd (Leeds, UK) (personal communication J Hippisley-Cox). In addition to providing risk estimates for each cancer based on the QCancer model, our tool gave summary information on best practice diagnostic pathways for each cancer based on Australian guidelines (2005) and Cancer Australia (2012).

### Simulated consultations

Six experienced actors from the Medical Education Unit, University of Melbourne, attended a 3-h training session to prepare them for two standardised clinical vignettes. One case was designed as a ‘low-intermediate risk' ovarian cancer case, and the second as a more complex case in which risks were raised for more than one type of cancer (Table 1). Videos were reviewed for fidelity of the simulated consultations and feedback given to actors to ensure consistency among them.

General practitioners were familiarised with the QCancer risk tool by using it for two patient paper-based vignettes, the details of which they entered into the tool as part of their training in its basic use. These vignettes were different from those used in the simulated consultations. The GP then used the QCancer risk tool in two simulated consultations with the actors in their usual clinic room. After each consultation a semi-structured interview was conducted, including the GP and the actor, asking them to reflect on the consultation. All interviews were audiotaped and simulated consultations were video-recorded.

The interview guide was developed using the normalisation process theory (NPT) (May *et al*, 2009). NPT is a well-established sociological theory that helps to understand how complex interventions and new technologies become embedded within routine clinical practice. There are four core constructs within the NPT that formed the basis for the areas explored in our interviews and for data interpretation (Murray *et al*, 2010):

1. Coherence relates to how participants make sense of an intervention, whether they can identify a clear purpose, the perceived benefits of the intervention and whether these benefits are valued.
2. Cognitive participation relates to whether participants buy into the intervention, agree it is part of their work and are prepared to invest time and energy in it.
3. Collective action relates to how compatible the intervention is with existing work practices, whether it promotes or impedes their work and how it fits within an organisation.
4. Reflexive monitoring relates to how participants appraise the intervention after it has been in use, whether the effects of the intervention are clear and the potential to adapt the intervention on the basis of experience.

Ethics approval for this study was obtained from the University of Melbourne Human Research Ethics Committee (ID 1341203).

### Analytical approach

We analysed the following sources of data: baseline GP demographics obtained by the questionnaire; transcripts of audio-recordings of interviews; video-recording of simulated consultations; and screenshots of QCancer outputs created during the consultations. Data were managed with NVivo (version 10; QSR International, Victoria, Australia). Each transcript was open coded, whereby each phrase was analysed to create key categories. A coding framework was developed and modified during the analysis of subsequent interviews according to the emerging concepts. Relationships between different categories were identified by constant comparison between and within transcripts and by comparison with existing literature. Videotapes of the consultations were reviewed, and observations were integrated into the developing conceptual framework (Barbour and Kitzinger, 1999; Braun and Clarke, 2006). Authors PP-C, JW and JDE met regularly to discuss the coding framework and resolve any differences in interpretation. The key results were presented at a meeting of six of the GP participants who agreed with the themes identified.

## Results

We recruited 15 GPs, from a total sample approached of 29, before both researchers agreed that data saturation had been reached. Characteristics of the participants are summarised in Table 2. We present our findings within the four key constructs of the NPT.

### Coherence

General practitioners were able to make sense of the purpose of the QCancer risk tool, could identify the tasks needed to use it and, in general, could identify a range of potential benefits from the tool. For example, they found the tool helpful in alerting them to the possibility of the presence of more than one type of cancer in the context of patients with more vague, non-specific symptoms. For some GPs it helped to prioritise the order or range of investigations to consider. General practitioners also identified a potential benefit, either to reassure patients at low risk of cancer and potentially reduce over-investigation, or to prompt action in patients who were reluctant to be investigated for suspicious symptoms. In these circumstances the numerical risk information was seen as a potentially beneficial aspect of the tool.

‘Think the biggest use I got, because you presented with very constitutional symptoms that a lot of things could present with, this was useful for me to think what's the good next step and what should I think of. So I was thinking in my mind more bowel stuff, but I didn't go into the ovarian stuff particularly. This tool will direct me to think about that as well'. GP-A (Male; 62)

‘Yes, I must admit ovarian didn't come so high up, I had already fixated on the bowel stuff, which maybe I shouldn't have. This really said hey, consider ovarian as well. So again that was useful, yes'. GP-B (Male; 46)

‘They'll come in, they would have seen their naturopath and they would have seen their acupuncturist, and they're coming back in just for reassurance and I'll say okay, you've done all those things, let's just put your numbers in and let's make sense of this, and then we'll see what tests we have to do' GP-C (Female; 60)

‘…it probably is a tool to encourage somebody to do some tests when they need to do tests'. GP-D (Male; 27)

‘I felt that sometimes when people come in concerned about ovarian cancer they want the full gamut of tests from the very beginning and I know that it is going to be expensive and stressful for them and probably not turn up much; they might be sweating over this gamut of tests over the next six weeks and then waiting to see me etc. So this way I could—I felt that—it depends on how happy you are with statistics too and you saw I struggled over that, trying to explain statistics to you in a usable form'. GP-E (Male; 56)

However, for some GPs there was confusion about the intended purpose of the tool, with some considering it to have a role in promoting cancer screening and discussions about cancer prevention and lifestyle factors rather than only for use in people with symptoms. The information about clinical recommendations was not always accessed by GPs, and, even when it was, GPs would still provide clinical advice outside these clinical guidelines.

‘If I had a patient here who I was particularly concerned about, but this was not on his radar at all and this box comes up and says screen for lung cancer. I'd be really worried and so to me, it's more I would like to have things pop up saying patient's eligible for bowel cancer screening, mammogram. I think that—Then if I click on [QCancer], it says, look, a woman of 45, put in the family history. This is her risk. This is how she should be screened'. GP-A (Male; 62)

‘Certainly with the cardiovascular risk assessment tool, that was always designed to do with the patient, because what you're trying to do is make them make the change. So if the design of this is actually to assist the patient to make lifestyle changes—so say for instance you went through all this. Your chest X-ray is perfectly normal. Your cough settles. Yes you still got airways disease, but we don't think you're actually a high risk of cancer, we don't think it's there. I still have to try and convince you to stop smoking, to exercise, to lose weight, to do all those things. So it should be used as a relationship tool'. GP-F (Female; 50)

‘Well, I'm gonna do the investigations no matter what. But what this is doing is if ovarian cancer is one in a thousand, I would've gone bowel first.' GP-G (Male; 66)

### Cognitive participation

General practitioners recognised the fact that they have an important role in diagnosing cancer and investigating patients with suspicious symptoms, but the low prevalence of cancer in primary care led to low levels of suspicion of cancer. This meant that they would rarely consider using the QCancer risk tool in their routine consultations, especially as cancer is only one of several possible diagnoses to consider in people with non-specific symptoms. General practitioners reflected that they felt obliged to use the tool for the purpose of the study and their suspicion of cancer was unnaturally heightened. Despite this, in three cases GPs conducted the consultation and chose not to use the tool at all. The QCancer risk tool was often perceived as a research tool and peripheral to their main task of delivering patient care.

‘My experience with ovarian cancer is probably—I can think of four in the last 30 years. So I wouldn't be thinking of it'. GP-H (Male; 59)

‘And the concept is we're doing a study on a cancer tool, you sort of—you're predisposing. That's in your mind'. GP-I (Female; 53)

Younger, less experienced GPs recognised that the QCancer risk tool could be incorporated into their work and might be beneficial to reduce the risk of missing a cancer diagnosis and increase their confidence in their clinical decisions. However, more experienced GPs were less likely to agree with the underlying concept and continued to maintain strong belief in their clinical intuition. In particular, experienced GPs often formulated clinical recommendations, which they discussed with the patient before even using the tool. If this was contradicted by the tool, they would have greater faith in their clinical judgement. This was the case even when there was a large discrepancy between their clinical assessment and the QCancer risk estimates, which led to mistrust of the risk model rather than questioning their clinical intuition. General practitioners would often order the investigation they initially decided on if it was easily accessible but would include the possibility of further investigations as recommended by the tool if the initial investigations showed nothing. For example, a CT may be ordered first despite QCancer demonstrating a lower risk of lung cancer compared with bowel cancer, because of ease of access to radiological investigations in Australian general practice. Nonetheless, the QCancer tool would prompt the clinician to consider a referral for colonoscopy if the CT was clear.

‘Normally I'd get a few investigations, get the results back and then based on that say do we need to do something, or I refer this on based on that. But I guess if I have a calculator saying it's higher risk, it might prompt me to make a referral to a specialist a bit earlier.' GP-J (Female; 31)

‘Because chest X-rays are easy and the gastrocope is gonna take me a couple of weeks to organise. So there's a logistical issue. Chest X-ray we can get a photo in two seconds and that's gonna happen now.' GP-B (Male; 46)

### Collective action

The most significant implementation challenges were identified within this NPT construct. Although the actors were trained to present the clinical history in a consistent way, there was a surprising degree of variation in the interpretation of GPs. Consequently, there was large variation in the symptoms and risk factors that were recorded in the QCancer risk tool and subsequent wide range of estimates of cancer risk for the same clinical vignette. For example, the risk outputs for case 1 for ovarian cancer ranged from 3.2 to 33.3% between GPs. For case 2, risk estimates for the most likely diagnosis (that is, pancreatic cancer) ranged from 8.7 to 12.0% and for lung cancer from 9.4 to 40.5%. General practitioners varied between entering, for example, either ‘change in bowel habits' or ‘constipation' only, or both, for a patient reporting constipation. Similarly, some GPs would interpret bloating as ‘abdominal swelling', whereas others would select ‘abdominal pain'.

The overall interface of the software was felt to be easily navigable and quick to use, but there were challenges in introducing the tool into consultation. Moreover, the focus specifically on cancer meant that other non-malignant diagnoses were not considered and the consultation became concentrated only on the issue of cancer rather than on broader assessment and management of the patient's symptoms. Introducing the whole concept of ‘cancer' into the consultation generated some anxiety for both the doctor and the simulated patient.

‘I then think because cancer, I think, is far more emotive than a risk of having a heart—You see, this is an absolute risk of having a cancer, but when you talk to people about their long term risk of having a heart attack and I'm talking about some people one in five, one in four over the next five years, and it doesn't worry them because everyone survives heart attacks these days. And everyone has a heart attack, but cancer's still got a really bad rep even though we treat it so well.' GP-K (Male; 48)

The main risk output, which lists probabilities of the full range of cancers covered by the QCancer model, was felt to be too ‘confronting' to use in a consultation, a term used by many GPs in this study. This was especially the case when variable interpretation of the history led to ticking multiple symptom boxes in the tool and subsequent overestimation of cancer risks. Sudden presentation of these high cancer risks led to loss of control of the consultation by the GP who then felt compelled to spend the rest of the consultation trying to reassure the patient.

‘And I thought, ‘Wow! Patients coming with these tummy pains,' even if I'm thinking, ‘She's got a 10% chance,' to actually think, ‘Wow! Listen, you got a 38% chance of cancer.' General public are very—I would find that confronting and the general public have a shocking record at understanding risk.' GP-B (Male; 46)

‘Yeah. If I start saying to them, ‘Oh god, your risk of having gastro-oesophageal cancer is one in three'… that seems way over the top, back pedalling from that, the consultation would've gone on for another 20 minutes or 30 minutes since I tried to explain to him, no. He doesn't have it'. GP-L (Male; 53)

Their experiences of the challenges of using the tool within the consultation led to GPs discussing alternative approaches to implementation, which would be more compatible with their work practices. Some GPs were interested in exploring the possibility of patients using the tool in the waiting room as a form of screening for symptoms in advance, allowing GPs to be better prepared, and avoiding the potentially confrontational aspects of using the tool with the patient.

‘Yeah. Maybe even some very, very busy general practices would be using other, maybe not as qualified staff to do it. That—some of the nurses would be excellent at it, but some of the nurses I think would need extra training'. GP-A (Male; 62)

‘If you have it going in the waiting room, people can download it in the waiting room on to their iPhone while they're waiting for the doctor and say, by the way I did the screening app' GP-K (Male; 48)

### Reflexive monitoring

General practitioners were able to reflect on their immediate experience of using the tool in terms of its impact on the consultation and their decision making. However, the low prevalence of cancer in primary care meant that, at an individual GP level, they would be unlikely to perceive the impact of the tool on their cancer diagnostic ability.

Several recommendations were made on how to adapt the tool based on their initial experience, predominantly centred around the confrontational aspects of the risk outputs. These included a traffic-light colour coding of risks (red, amber and green) rather than absolute numeric risks, or presenting diagnostic guidance as the primary output with secondary access to the numeric risks if needed.

‘The first thing that comes up after calculate probably needs to be really much patient-directed rather than doctor-directed…It needs to be fairly non-confrontational from the patient. And then maybe not even using the word risk, you know, that's where the colour coding might come in. You know if you saw something in green, I think as patient, you probably think, ‘That's pretty—that's not too bad.' And maybe you know, if it's not green, you know, yellow might just be warning... And you just have it worded like in normal colour, just saying, you know, strongly recommend that you have this test and that test done. And then the doctor would know, ‘Well, hang on.' We would know if that's not good. Yeah, I think that's a pretty strong recommendation. The patient wouldn't necessarily get that feeling. But we would be able to say, ‘Look, it recommends that you have this test done. What about we do that test?' GP-B (Male; 46)

The current version of the tool was not integrated into the general practice clinical software, but this was seen as an important adaptation if it were going to be used. Suggestions also included linking the tool within the clinical software to investigations in order to affect decisions at the time of ordering specific tests. The interviews specifically explored the alternative implementation strategy of running the QCancer model as an audit across the practice population to identify patients who may require further investigation when they next visited. This was not well received by the majority of GPs who expressed concerns about ‘prompt fatigue' with too many electronic reminders already occurring in their clinical software. General practitioners generally did not have any consistent internal auditing processes to determine the success or otherwise of the QCancer risk tool, which would be important if it were to be used in an ongoing way.

I'm wondering if this fits better in the bit where you're ordering tests, like when your diagnostic bit because I'm just wondering because it seems to be that's where it's really helping me. ‘Cause probably, I could tell you now, I would have done a blood test on this guy and I should have, but I would've forgotten to do an amylase, for example, because he's got a big pancreas to his right and probably without freaking him out, well, I would've done a haemoglobin..' GP-M (Male; 60)

## Discussion

This is the first study to our knowledge to explore in-depth the challenges of implementing a cancer diagnostic risk assessment tool in general practice using simulated consultations. We identified some key issues for consideration about the clinical utility of these types of tools, especially if they are to be used within a general practice consultation.

### Strengths and limitations

Our sample of 15 doctors was purposively selected to represent a range of skills, opinions and clinical experience. Although our sample included GPs with many years of experience, we also recruited one academic registrar and several more recently qualified GPs. Some of the themes that arose in this study are common to those reported in a qualitative study of the Macmillan eCDS pilot tool (Dikomitis *et al*, 2012). It is possible, therefore, that our findings may be transferable to other GPs internationally. However, qualitative research does not attempt to generalise but to provide a deeper understanding of phenomena and generate results of high validity (Silverman, 1993). Although our participants used the risk tool in specific scenarios, the use of simulated patients has been shown to predict actual clinical performance (O'Hagan *et al*, 1986). We used a range of data sources to triangulate our findings; two experienced researchers conducted every interview, and the actors were highly skilled at providing consistent information in each consultation. The use of the influential theoretical perspective of NPT to collect and analyse our data is a significant strength; it provides a framework for the explicit consideration of the implementation challenges of a complex intervention and can help explain why electronic clinical decision support tools may not have the expected outcomes.

### Key findings

Although GPs in this study could identify potential benefits of the QCancer risk tool, the perception that cancer was not often considered by GPs as a diagnosis meant they would rarely consider using it in a consultation. This contrasts with the findings from the Macmillan eCDS pilot (Dikomitis *et al*, 2012) and may reflect sustained policy efforts in the UK to raise awareness about early cancer diagnosis that have not been made in Australia. We also found that clinical experience and belief in clinical intuition were determinants of tool use. Although clinical intuition may be useful in assessing people with symptoms suggestive of cancer (Hjertholm *et al*, 2014), it is still prone to significant biases. The Macmillan pilot found that the interactive risk calculator, similar in design to our tool but based on a different risk model, was not used often during consultations (Hamilton *et al*, 2013). Our findings shed important new light on why this may be.

General practitioners found the tool difficult to introduce into the workflow of the consultation and were reluctant to use the tool to avoid revealing potentially confronting information about cancer risks. Previous studies of family history-based cancer risk assessment tools have also shown the potential to ‘lose control of the consultation' if the risk information is presented too explicitly (Emery *et al*, 2000). Perhaps our most interesting and novel finding is the variable interpretation of the patient's standardised symptoms that led to widely different cancer risk assessments. Different boxes in the symptom checklist were scored for the same pattern of symptoms. This was surprising given that many of our GPs were very experienced, but we observed this phenomenon across younger and older doctors. A review of the videos confirmed that this was not due to differences in the ways actors presented the history. Previous research has identified the importance of careful interpretation of the term ‘bloating' when assessing risk of ovarian cancer (Bankhead *et al*, 2008). This issue may extend to other key symptoms and how they are interpreted in relation to symptom checklists in risk assessment tools. This finding could be a major problem in terms of the reliability of risk assessment with such tools across not only cancer but also other non-malignant conditions.

Our tool differed in some important ways from the Macmillan eCDS: the interface was developed specifically for this project; it was not integrated into the clinical software; and it offered specific diagnostic advice. It was more similar to the existing QCancer online tool (www.qcancer.org). Some of our findings may be specific to our tool, but we believe that many are relevant to the implementation of cancer diagnostic risk tools in general.

## Conclusion

Cancer diagnostic decision support systems are still in their infancy, as are the best methods to implement them so that they can be used routinely and safely. This study highlights the challenges in implementation that should inform the interpretation of existing studies of eCDS and future randomised trials aimed at testing their effects on early cancer diagnosis.

## Figures and Tables

**Table 1:** Vignettes for simulated consultations

**Case one**
You are a 55 year old woman and you have had no serious illnesses in the past.
For the past few weeks you have felt bloated and that your tummy seemed bigger than usual. You feel a bit constipated but you have had no bleeding or any other bowel symptoms. Your weight is steady and you have felt a bit more tired than usual but you have been working longer hours recently. You have also been getting some indigestion after meals. You have no other symptoms.
Your mother had breast cancer in her 60s and there is no other relevant family history.
You have never smoked and drink a glass of wine 2-3 times per week. You are married and your children are away at university.
You weigh 61 kg and are 162 cm tall.
QCancer risk estimates: ovarian cancer 1.72% colorectal cancer 0.27%
**Case two**
You are a 65 year old man who has had a bad cough for the last month which won't go away. You have not coughed much up and in particular you have not coughed up any blood. You saw another GP about this a couple of weeks ago and the antibiotics he gave have made no difference.
You have also been getting indigestion and pain in your stomach (centrally just below your ribs) and have taken a few indigestion tablets from the pharmacy for it. You have lost a few kilos recently as your trousers aren't as tight as usual. Your bowels are normal and you don't have any other symptoms.
You have a history of diabetes for which you take tablets every day and emphysema (you are on 2 puffers)
You have smoked 10-15 cigarettes a day for many years and have a couple of beers each night.
Your wife died a few years ago of breast cancer and you live alone. Your parents and grandparents all died of ‘old age' in their 80s.
You weigh about 105 kg and are 180 cm tall.
QCancer risk estimates: pancreatic 18.68% gastro-oesophageal 11.98% lung 9.70%

**Table 2:** Participants' baseline characteristics

**Characteristics**	***n* (%)**^a^
Age, years^b^	53 (27–66)
Gender, female	5 (33.3)
Number of years in general practice^b^	25 (0.6–36)
Hours worked in an average week^b^	27 (5–40)
Practice location, metropolitan	12 (80.0)
Specialisation in an area of general practice	
Type of specialisation^c^	
*Prostate cancer*	1 (9.1)
*Rural health*	1 (9.1)
*Mental health*	3 (27.3)
*Chronic disease management*	1 (9.1)
*Musculoskeletal*	1 (9.1)
*Complementary medicine*	1 (9.1)
More than one type of specialisation	3 (42.9)
Postgraduate qualifications	12 (80.0)
More than one qualification	6 (50.0)
Number of GPs working in practice:	
Full-time	3 (1, 3)
Part-time	6 (4, 8)
Informal cancer training past 12 months	5 (33.3)
Type of training	
RACGP course	2 (40.0)
Conference workshop	1 (20.0)
Seminar	2 (40.0)
Other professional activities aside from clinical	
None	1 (6.7)
Teaching medical students only	3 (20.0)
Teaching students and registrars	4 (26.7)
Teaching and research	6 (40.0)
Conducting research only	1 (6.7)
Currently use any risk calculator tool(s)	14 (93.3)
AusDiab and cardiovascular risk tool	4 (28.0)
Cardiovascular risk tool	8 (57.1)
AusDiab, cardiovascular risk tool, other^d^	1 (7.1)
Cardiovascular risk tool and other	1 (7.1)

Abbreviations: FRAX=fracture risk assessment tool; GPs = general practitioners; K10=Kessler Psychological Distress Scale; PHQ2=Patient Health Questionnaire 2; RACGP=Royal Australian College of General Practitioners.

a Unless specified.

b Median (range).

c Total do not add up to *n*=7 as some GPs have more than one type of specialisation.

d FRAX, NZ cardiovascular risk tool, PHQ2, KIO, UK.
